# A combined microfluidic-transcriptomic approach to characterize the extravasation potential of cancer cells

**DOI:** 10.18632/oncotarget.26306

**Published:** 2018-11-16

**Authors:** Simone Bersini, Agnes Miermont, Andrea Pavesi, Roger Dale Kamm, Jean Paul Thiery, Matteo Moretti, Giulia Adriani

**Affiliations:** ^1^ Cell and Tissue Engineering Laboratory, Istituto Ortopedico Galeazzi, Milano, Italy; ^2^ BioSystems and Micromechanics IRG, Singapore-MIT Alliance for Research and Technology, Singapore; ^3^ Institute of Molecular and Cell Biology (IMCB), Agency for Science, Technology and Research (A*STAR), Singapore; ^4^ Department of Biological Engineering, Massachusetts Institute of Technology, Cambridge, MA, USA; ^5^ Yong Loo Lin School of Medicine, Department of Biochemistry, National University of Singapore, Singapore; ^6^ Regenerative Medicine Technologies Laboratory, Ente Ospedaliero Cantonale, Lugano, Switzerland; ^7^ Swiss Institute for Regenerative Medicine, Lugano, Switzerland

**Keywords:** microfluidics, microarrays, cancer cell extravasation, tumor microenvironment, organ-specific metastasis

## Abstract

The reciprocal interaction between circulating tumor cells (CTCs) and tissue-specific cells is influential for the progression of metastases. In particular, the process of extravasation relies on the complex cross-talk between cancer cells and other cellular players such as the endothelium and the secondary tissue. However, most *in vitro* studies only focus on one heterotypic cell-cell interaction and often lack of physiological relevance. In this project, we investigated both CTC-endothelium and CTC-secondary site interactions during cancer cell extravasation. We first used a microarray analysis of extravasated MDA-MB-231 breast cancer cells to identify key markers involved in extravasation. Then, we developed a tri-culture microfluidic platform combining cancer cells, endothelium and a bone-mimicking (BMi) microenvironment to assess how organ tropism influences the extravasation potential of cancer cells from different tissues. Through the microarray analyses of extravasated cancer cells we found that extravasation is associated with upregulation of late-metastatic markers along with specific proteases, such as matrix metalloprotease (MMP), a-disintegrin and metalloprotease (ADAM) and a-disintegrin and metalloproteinase with thrombospondin motifs (ADAMTS) family members, which are all involved in endothelium glycocalyx shedding. Through the microfluidic extravasation assay, we found that the bone-like microenvironment increased invasion and motility of breast, bladder and ovarian cancer cell (MDA-MB-231, T24 and OVCAR-3). Among the three cell types, ovarian cancer cells presented the lowest migration rate and bladder cancer cells the highest, hence recapitulating their different level of bone tropism observed *in vivo*. Taken together, our results shed light on the importance of intercellular communication between CTCs and other non-tumor cells essential for promoting cancer cell extravasation.

## INTRODUCTION

Tumor metastasis is the primary cause of mortality among cancer patients, mainly caused by the resistance of disseminated cells to most current therapeutic agents [1]. One of the critical steps in cancer metastasis is the extravasation of circulating tumor cells (CTCs) from the blood microcirculation to a distant organ. Extravasation relies on the cross-talk between cancer cells and their microenvironment, which includes the endothelium and the tissue of the secondary site. The first fundamental heterotypic interaction during this process happens with the adhesion (or physical trapping) of CTCs to the microvascular wall allowing cancer cells to transmigrate into the tissue parenchyma. Under normal physiological conditions, endothelial cells (ECs) are coated with a thin carbohydrate-rich structure called glycocalyx. Biochemically, it is composed of proteoglycans, glycoproteins and glycolipids associated with the plasma membrane [2]. The EC glycocalyx plays a protective role at the cell surface. Its main functions are as a vascular permeability barrier, mechano-sensor of hydrodynamic shear blood forces and regulator of adhesive interactions between stimuli - such as enzymes, cytokines or adhesion molecules - and the endothelium. Since the EC glycocalyx is five times thicker than an average membrane receptor [3], its presence is essential to maintain optimal receptor-ligand interactions. In addition, the thickness of the glycocalyx acts as a barrier against external stress and could impede the process of cell transmigration [4]. Several processes are known to remodel and affect the glycocalyx, such as inflammation or changes in hemodynamic shear stress. During those events, degradation of the glycocalyx is triggered by exposure of the endothelium to extracellular proteases. Although matrix metalloproteases (MMPs) have been well characterized in the shedding of syndecans [4], there is increasing evidence that other proteases are also involved such as thrombin, plasmin, elastase and members of the a-disintegrin and metalloprotease (ADAM) family [5]. CTC adhesion and transmigration have been observed to disrupt heparan sulfate (HS), the most abundant glycosaminoglycan (GAG) in the EC glycocalyx [6]. However, the direct action of CTCs on the EC glycocalyx, notably regarding the proteases involved, has never been clearly demonstrated.

Extravasation is not only determined by the interplay between CTCs and the endothelium, since cross-talk between cancer cells and targeted secondary tissues also significantly contribute to cancer cell dissemination [7]. Consequently, certain tumors will preferentially invade one or several specific secondary organs. This “organ tropism” of metastasis is thought to stem from several factors, such as an organ-specific circulation pattern, as well as reciprocal interactions between the host microenvironment and the cancer cells [8]. Preferential secondary sites vary depending on the tumor type. Bladder and breast cancer mainly form metastasis in bone, liver and lung [9, 10], while ovarian cancer frequently metastasizes in the peritoneal cavity [11]. Among the most common site of metastasis, bone is one of the most permissive environments for colonization and bone metastases are a major cause of cancer-related pain associated with secondary pathologies, such as hypercalcemia or pathologic fractures [12].

The process of CTC extravasation has been studied *in vivo* using intravital videomicroscopy of transfected tumor cells in mice [13]. These models can capture the complexity of the metastatic process; however, they are often limited in terms of their ability to probe and quantify specific mechanisms. *In vitro* models provide better control of different biological parameters, use small fluid volumes and facilitate high-resolution real-time acquisition of data compared to traditional animal models [14, 15]. Furthermore, *in vitro* microfluidic systems are powerful tools for reductionist studies of the different steps of metastasis [16–20], notably to recapitulate extravasation [7, 21, 22]. These models also present the advantage - compared to standard *in vitro* or *in vivo* studies - to visualize and quantify the interactions of multiple cell types, either in 2D [23] or 3D [24–27]. Despite exhaustive studies on cancer cell extravasation using *in vitro* systems, none have looked simultaneously at the cross-talk taking place among cancer cells, the microvascular wall and the secondary metastatic site. In this study, both standard Transwell assays and a microfluidic *in vitro* model have been used to analyze the impact of cell-cell interactions between cancer cells, ECs and osteo-differentiated (OD) human bone marrow-derived mesenchymal stem cells (hBM-MSCs) on the extravasation ability of cancer cells. In particular, we have demonstrated that extravasated cancer cells upregulate genes involved in glycocalyx shedding and that bone tropism helps to mediate the extravasation of cancer cells from different primary tumors.

## RESULTS

Two different approaches were used to investigate the heterotypic intercellular interactions during the process of CTCs extravasation. The first approach combined Transwell assay and Affymetrix microarray analysis to study the impact of CTCs gene expression on metastatic progression and vascular barrier reorganization. In the second part, to further investigate the cancer cell extravasation beyond the interplay between cancer cells and endothelium, we decided to study the cancer cell transmigration across the endothelium in presence of a secondary tissue. For this purpose, we chose a microfluidic assay to mimic a bone-like environment and observe the organ-specific metastatic potential of three different cancer cell lines in a more physiological setting compared to the Transwell assay.

### Clear signature of cancer cells from microarrays data

**Figure 1:**
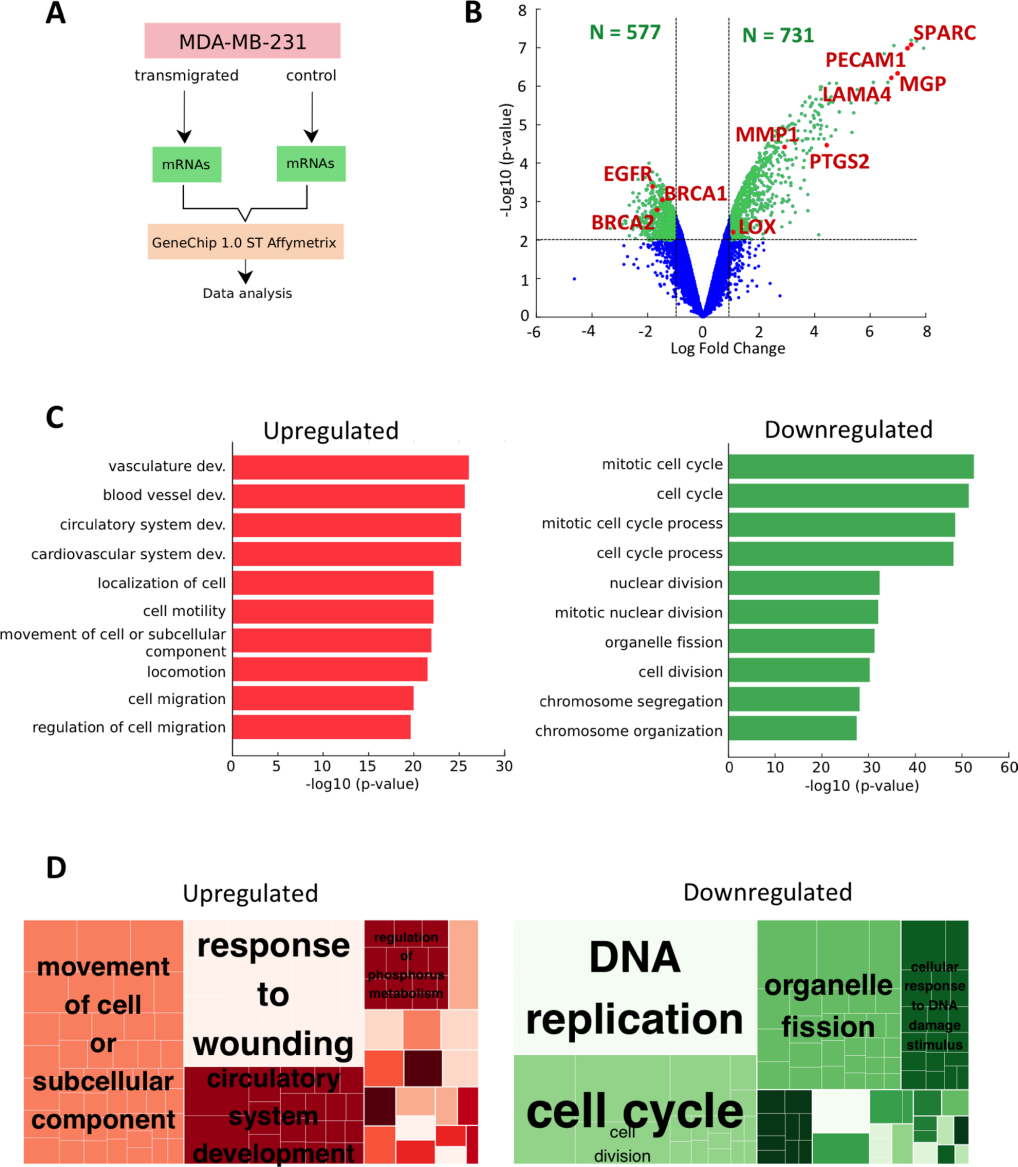
Genetic markers of metastasis progression from microarrays data (**A**) Schematic representation of the procedure to examine differential gene expression after cell extravasation. (**B**) Volcano plot of microarrays data. The data for all genes are plotted as the log2 fold change versus the negative log10 of the adjusted *p*-value. Thresholds are shown as dashed lines. The upregulated DEG (*p*-value < 0.01 and log FC > 1) and downregulated DEG (*p*-value < 0.01 and Log FC < −1) are represented as green dots. The red and labeled dots correspond to markers of late-metastatic stage. (**C**) The top 10 Gene Ontology (GO) enriched Biological Processes (BP) terms for the DEG of trans-migrated MDA-MB-231 cancer cells. The respective red (upregulated terms) and green (downregulated terms) bar charts represent the −log10 (*p-*value) of the GO term. (**D**) REVIGO treemaps of over-represented GO terms processed (*p*-value < 0.01) for upregulated (left) and downregulated (right) BP lists. Each large rectangle, called supercluster, represents a cluster of loosely related GO terms (small rectangles) that are displayed in different colors. The size of the rectangles reflects the corrected *p*-value (i.e. larger rectangles represent the most significant GO terms).

In order to analyze the alterations of transcriptome expression associated with cancer cell extravasation, we collected RNA samples from MDA-MB-231 breast cancer cells after having, or not, transmigrated through an endothelial monolayer. We then performed a global gene expression profiling using Affymetrix Human GeneChip 1.0-ST arrays (Figure 1A) and analyzed the differentially-expressed genes (DEGs) being either significantly upregulated (*p*-value < 0.01; log fold-change >1) or downregulated (*p*-value < 0.01; log fold-change <−1) in the transmigrated cells compared to the control cells. We obtained 731 upregulated and 577 downregulated DEGs (Supplementary Table 1). Among the ten most significant upregulated DEGs, we identified four genes with a clear role in metastatic progression and invasiveness (Figure 1B and Table 1). Two of these genes are the extracellular matrix (ECM) glycoproteins SPARC (secreted protein acidic and rich in cysteine) and MGP (matrix-gla protein) which have been previously described to promote cancer cell migration, invasiveness and increase tumor cell ability to form lung metastases [28]. Furthermore, these two markers play a direct role on endothelium integrity. Indeed, secreted SPARC by melanoma cells promotes vascular permeability [29] and tumor cells expressing MGP present an increased adhesion ability to the endothelium [28]. The two other genes identified are platelet endothelial cell adhesion molecule 1 (PECAM1) and laminin subunit alpha (LAMA4). PECAM1 is involved in the regulation of late-stage metastatic progression [30] while LAMA4, which encodes the secreted alpha chain isoform protein laminin-α4, is associated with metastatic progression and increased metastatic colonization of the lungs [31].

**Table 1:** Top 10 genes significantly up- or down-regulated in transmigrated MDA-MB-231 cancer cells

Symbol	Description	Fold change	*P*-value
***Upregulated***			
MMRN1	multimerin 1	7.916801137	1.01E-07
CALCRL	calcitonin receptor-like	7.656794428	6.55E-08
ADGRL4	adhesion G protein-coupled receptor L4	7.476770645	6.43E-08
SPARC	secreted protein, acidic, cysteine-rich (osteonectin)	7.473333422	8.32E-08
PECAM1	platelet/endothelial cell adhesion molecule 1	7.323915843	1.05E-07
MGP	matrix Gla protein	6.974442274	4.56E-07
FAM198B	family with sequence similarity 198, member B	6.850897183	8.79E-08
LAMA4	laminin, alpha 4	6.768113725	6.05E-07
TM4SF18	transmembrane 4 L six family member 18	6.64351334	8.01E-07
HHIP	hedgehog interacting protein	6.498144849	1.45E-07
***Downregulated***			
BIRC3	baculoviral IAP repeat containing 3	–3.328301245	0.004990633
TOM1L1	target of myb1 (chicken)-like 1	–2.905533729	0.003926993
CLGN	calmegin	–2.752323839	0.002335304
EHF	ets homologous factor	–2.668666334	0.007695197
ENPP1	ectonucleotide pyrophosphatase/phosphodiesterase 1	–2.64688309	0.001263539
ASNS	asparagine synthetase (glutamine-hydrolyzing)	–2.569326802	0.004995183
ERRFI1	ERBB receptor feedback inhibitor 1	–2.545947013	0.000597849
BUB1	BUB1 mitotic checkpoint serine/threonine kinase	–2.538966706	0.003429176
HLA-DPA1	major histocompatibility complex, class II, DP alpha 1	–2.534508184	0.005219536
MYBL1	v-myb avian myeloblastosis viral oncogene homolog-like 1	–2.527185068	0.003191445

We then investigated which metastatic stage exhibits the strongest correlation to the change of expression associated with transmigrated cancer cells. Indeed, DEGs can be grouped into classes, corresponding to the stepwise sequence of events, from metastasis initiation to metastatic virulence functions [32]. Referring to these metastatic phases, the upregulated DEGs solely correlated with the second-to-last stage, called “metastasis progression function”, which consists of extravasation, survival and re-initiation. Those genes are prostaglandin G/H synthase 2 (PTGS2), MMP1 and lysyl oxidase (LOX). The two first DEGs are part of a group of genes mediating extravasation of CTCs into the pulmonary parenchyma, while LOX acts on ECM proteins to promote metastatic niche formation [33]. From the downregulated DEGs list, we found only genes belonging to the first stage named “tumour initiation functions” and consisting of growth, survival, progenitor-like state and genomic instability. Those genes are the epidermal growth factor receptor (EGFR) and the breast cancer genes BRCA1 and BRCA2 (Figure 1B and Table 2). Taken together, our results suggest that the process of transmigration triggers a shift of expression of CTCs to a more virulent cellular state, where genes involved in the early stage are either switched or switching off while promoting the expression of genes involved in the late-metastatic stage.

**Table 2:** DEG known to be associated with metastatic progression (top) and glycocalyx degradation (bottom) in transmigrated MDA-MB-231 cancer cells

Symbol	Description	Fold change	*P*-value
***Metastasis progression***			
PECAM-1	platelet/endothelial cell adhesion molecule 1	7.323915843	1.05E-07
SPARC	secreted protein, acidic, cysteine-rich (osteonectin)	7.473333422	8.32E-08
MGP	matrix Gla protein	6.974442274	4.56E-07
LAMA4	laminin, alpha 4	6.768113725	6.05E-07
PTGS2	prostaglandin-endoperoxide synthase 2	4.451044578	3.42E-05
MMP-1	matrix metallopeptidase 1	2.925706769	3.79E-05
LOX	lysyl oxidase	1.064927102	0.006146137
EGFR	epidermal growth factor receptor	1.831665534	0.000396237
BRCA1	breast cancer 1, early onset	1.467999528	0.000897793
BRCA2	breast cancer 2, early onset	1.667288455	0.001614941
***Glycocalyx degradation***			
MMP-1	matrix metallopeptidase 1	2.925706769	3.79E-05
MMP-2	matrix metallopeptidase 2	3.755385959	0.001684929
MMP-10	matrix metallopeptidase 10	2.423435742	0.002273505
MMP-16	matrix metallopeptidase 16 (membrane-inserted)	2.662162679	1.71E-05
ADAM23	ADAM metallopeptidase domain 23	2.569506482	5.45E-05
ADAMTS1	ADAM metallopeptidase with thrombospondin type1 motif, 1	2.291032254	0.001276709
ADAMTS6	ADAM metallopeptidase with thrombospondin type 1 motif, 6	1.35536668	0.000572073

Using the gene functional classification tool DAVID, the DEGs from both upregulated and downregulated lists have been categorized into functional groups and ranked based on their lowest *p*-values. The most significant biological processes from each upregulated and downregulated list are shown on Figure 1C and Table 3. To reduce redundancy while facilitating visualization of the most significant biological processes (BPs), the full list of upregulated and downregulated Gene Ontology (GO) terms (*p*-value < 0.01) were loaded and processed in REVIGO generating hierarchical treemaps (Figure 1D). In the upregulated list, three major GO superclusters were detected and consist of movement of cell or subcellular components, response to wounding, and circulatory system development (Figure 1D left panel). In the downregulated list, the enriched BPs are clustered into three dominant processes: DNA replication, cell cycle and organelle fission (Figure 1D right panel). Those results are consistent with the most significant BPs of each DEGs list (Figure 1C and Table 3). Therefore, movement of cell and DNA replication are the major GO superclusters for respectively the upregulated and downregulated DEGs list.

**Table 3:** Functional enrichment of the 15 most significant up- and down-regulated GO biological processes in transmigrated MDA-MB-231 cells

Accession	Biological process	Count	*P*-value
***Upregulated DEG***			
GO:0001944	vasculature development	81	9.87E-27
GO:0001568	blood vessel development	78	2.81E-26
GO:0072359	circulatory system development	101	7.04E-26
GO:0072358	cardiovascular system development	101	7.04E-26
GO:0051674	localization of cell	117	7.79E-23
GO:0048870	cell motility	117	7.79E-23
GO:0006928	movement of cell or subcellular component	140	1.33E-22
GO:0040011	locomotion	126	3.50E-22
GO:0016477	cell migration	105	1.19E-20
GO:0030334	regulation of cell migration	76	2.62E-20
GO:0048514	blood vessel morphogenesis	63	5.45E-20
GO:0040012	regulation of locomotion	80	1.03E-19
GO:0009653	anatomical structure morphogenesis	170	3.99E-19
GO:2000145	regulation of cell motility	77	4.42E-19
GO:0051270	regulation of cellular component movement	80	1.35E-18
***Downregulated DEG***			
GO:0000278	mitotic cell cycle	122	3.42E-53
GO:0007049	cell cycle	156	4.34E-52
GO:1903047	mitotic cell cycle process	113	3.26E-49
GO:0022402	cell cycle process	137	8.58E-49
GO:0000280	nuclear division	76	5.16E-33
GO:0007067	mitotic nuclear division	66	1.03E-32
GO:0048285	organelle fission	77	6.20E-32
GO:0051301	cell division	73	6.46E-31
GO:0007059	chromosome segregation	55	9.24E-29
GO:0051276	chromosome organization	100	4.37E-28
GO:0006259	DNA metabolic process	85	5.82E-24
GO:0098813	nuclear chromosome segregation	45	1.31E-22
GO:0000819	sister chromatid segregation	40	3.41E-22
GO:0044772	mitotic cell cycle phase transition	58	7.11E-22
GO:0006260	DNA replication	44	1.56E-21

### Upregulation of several extracellular proteases involved in endothelial glycocalyx rearrangement

**Figure 2:**
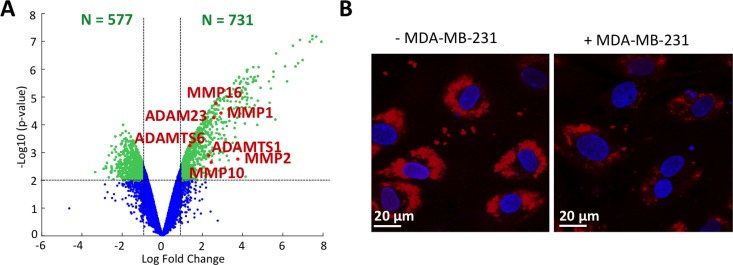
Glycocalyx degradation (**A**) Volcano plot of microarrays data. The red and labeled dots correspond to markers of glycocalyx degradation. (**B**) Endothelial glycocalyx labeled using lectin staining (TRITC) with (right) or without (left) the presence of MDA-MB-231 in the microfluidic channel. Cell nuclei are shown in blue (Hoechst).

We then explored the presence of endothelial glycocalyx degradation markers in the transmigrated MDA-MB-231 breast cancer cells. We observed that several MMPs, namely MMP1, MMP2, MMP10 and MMP16 are significantly upregulated in our dataset (Figure 2A). The role of MMP10 and MMP16 on the endothelium is unknown, however MMP1 and MMP2 are capable of respectively cleaving heparan sulfate proteoglycan syndecan-1 and chondroitin sulfate which are both constituents of the glycocalyx [34, 35]. We also observed a significant upregulation of cathepsin B, which is implicated in the loss of hyaluronan from the endothelial surface layer [36]. ADAM23 and the ADAMTS (a-disintegrin and metalloproteinase with thrombospondin motifs) 1 and 6 are also over-represented in our dataset. ADAMTS1 has an anti-angiogenic role, while the role of ADAMTS6 has not been defined but it is known to be dysregulated in breast carcinoma [37].

Finally, the endothelial glycocalyx was characterized through lectin staining in the endothelial channel of the microfluidic device to detect potential structural defects on the endothelial surface due to the presence of cancer cells (Figure 2B). We observed that lectin expression on ECs was reduced when MDA-MB-231 cells were present compared to ECs without cancer cells. Overall, our genetic analysis coupled with qualitative immunofluorescence results demonstrate that MDA-MB-231 cells have a direct negative impact on glycocalyx structure, with direct implications in cancer cell extravasation.

### Characterization of the vascularized BMi microenvironment

**Figure 3:**
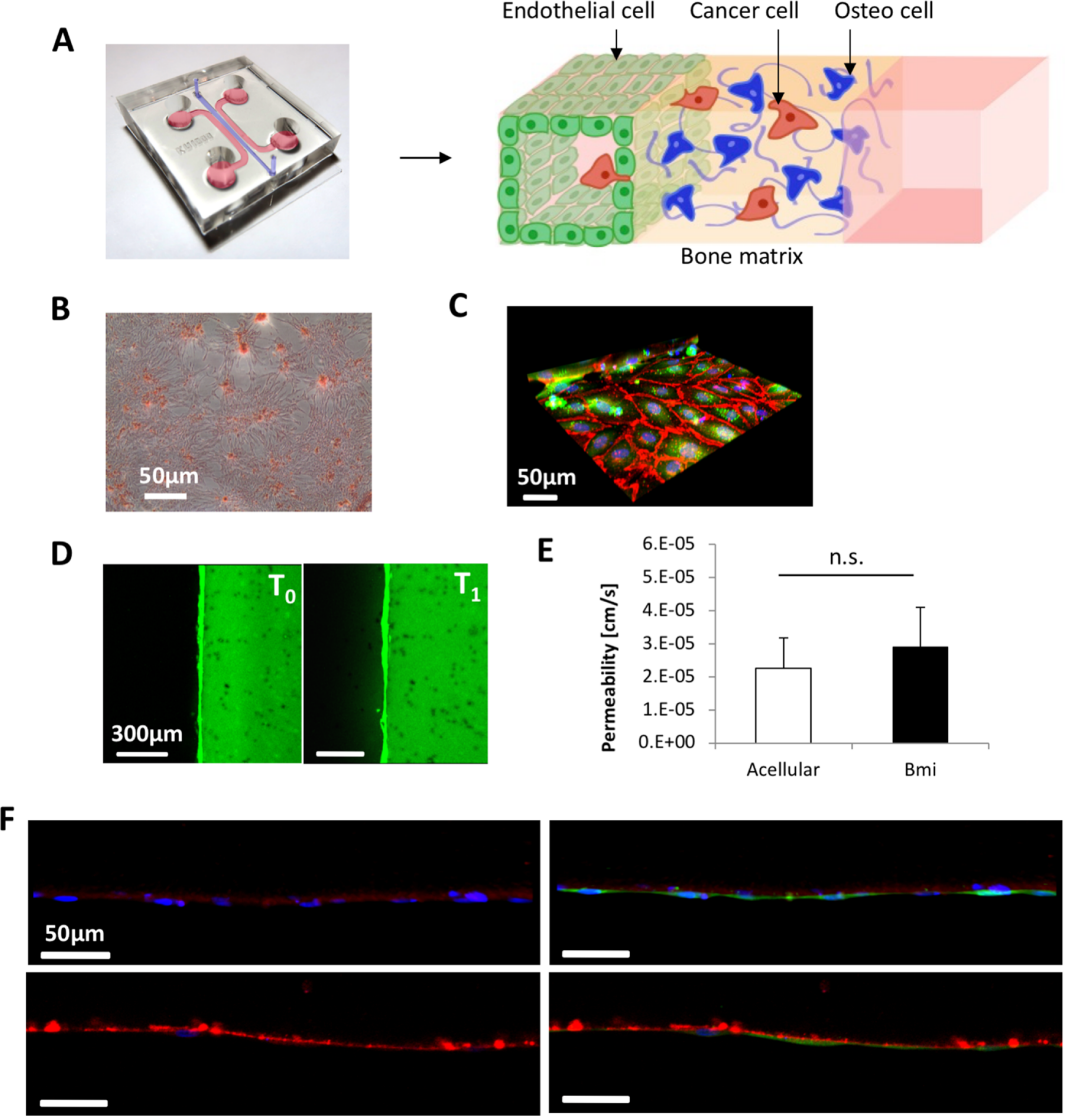
Characterization of the bone mimicking (BMi) microenvironment (**A**) schematic/photo of the tri-culture microfluidic device for cancer cell extravasation experiment toward a bone-like environment. (**B**) Alizarin Red-S assay showing deposition of calcium (red spots) by mesenchymal stem cells. (**C**) 3D reconstruction of confocal stack images showing vascular endothelial cadherins (red) highlighting the formation of adherens junctions between endothelial cells (green). Cell nuclei are in blue (Hoechst). (**D**) Green fluorescent dextran was injected in the media channels and endothelial permeability quantified comparing fluorescence intensity profiles at T_0_ (6 min) and T_1_ (8 min). These are representative images for the BMi matrix. (**E**) Permeability coefficients of dextran across BMi and acellular matrices. (**F**) Laminin (red in the upper images) and collagen IV staining (red in the lower images) demonstrating the presence of a mature basement membrane which supports the growth of the endothelial monolayer (green in the right images). Cell nuclei are shown in blue (Hoechst).

A BMi microenvironment was generated in the microfluidic device to characterize the effect of organ-specificity on the extravasation of different cancer cell lines (Figure 3A). As previously demonstrated, OD cells condition the ECM through naturally secreted proteins and spontaneously generate biological gradients [7]. Moreover, OD cells were shown to generate calcium deposits, thus confirming their ability to produce an early stage mineralization of the surrounding matrix (Figure 3B). The absence of post structures at the interface between matrix and media channels allowed the generation of a continuous endothelial monolayer throughout the whole channel length, thus significantly improving the previously developed BMi model [7], which presented a few breaks in the endothelium close to the PDMS posts. The tight connections between endothelial cells seeded in the media channel were confirmed by VE-cadherin staining (Figure 3C) and the endothelial permeability was then quantified through fluorescent dextran (70 kDa, 20 μg/ml, green) for both BMi and acellular matrices (Figure 3D shows a representative image of the permeability assay for the BMi matrix). Permeability values were computed based on the differences in the fluorescence intensity between two time points which were labeled T_0_ and T_1_. No significant differences were detected between these two conditions with values in the order of 10^−5^ cm/s (Figure 3E). The presence of a mature endothelium was instead highlighted by laminin and collagen IV deposition (Figure 3F). Taken together, these results demonstrate a functional vascularized BMi microenvironment which can be employed to study the organ-specific metastatic spread of different cancer cell lines.

### Cancer cell extravasation in BMi microenvironment: comparison between human breast, bladder and ovarian cancer cells

**Figure 4:**
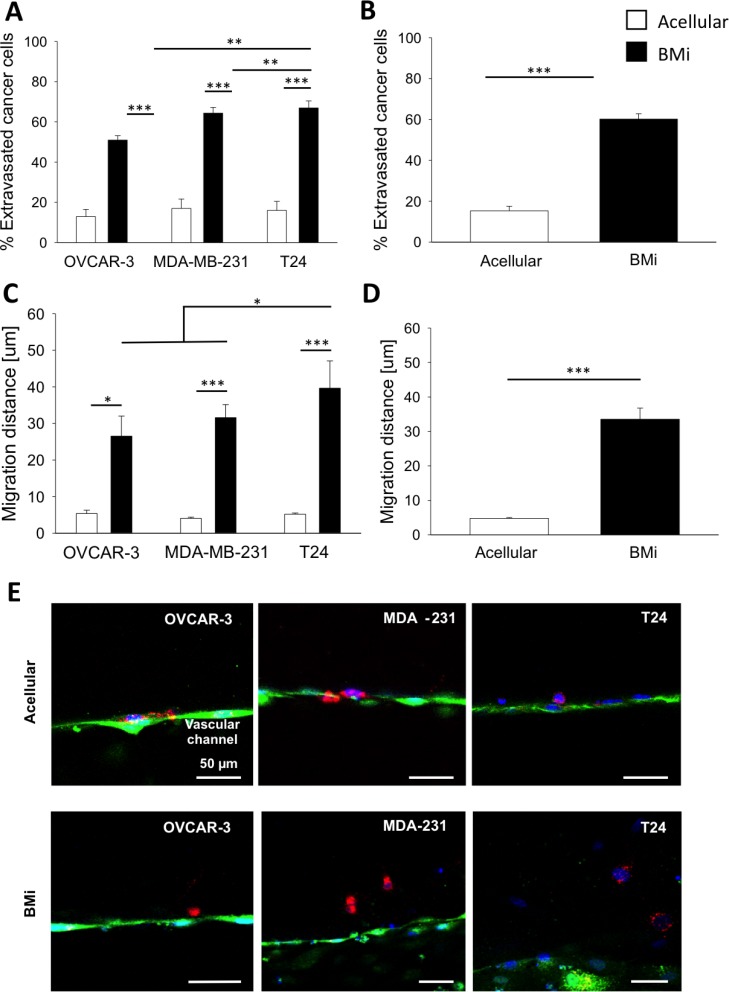
Extravasation and migration data (**A**) Bar plots of mean ± SEM of the percentage of extravasated cancer cells considering OVCAR-3, MDA-MB-231 and T24 cells in acellular or BMi microenvironment condition. (**B**) Bar plots of mean ± SEM of % of extravasated cancer cells combining the data for all the three cell types in either acellular or BMi microenvironment condition. (**C**) Bar plots of mean ± SEM of the migration distances from the endothelial layer for OVCAR-3, MDA-MB-231 and T24 cells in acellular or BMi microenvironment condition. (**D**) Bar plots of mean ± SEM of % of the migration distances from the endothelial layer for all the three cell types in either acellular or BMi microenvironment condition. Statistical significance was evaluated by a two-tailed *t*-test. ^*^=*p* < 0.05; ^**^=*p* < 0.01, ^***^=*p* < 0.001. (**E**) Representative images of the 3 different cancer cell types extravasated into the extracellular matrix in acellular (top panel) or BMi (bottom panel) microenvironment condition. Endothelial layer (green), cancer cells (red), cell nuclei (blue).

Cancer cell extravasation was investigated within BMi microenvironments and acellular collagen gels. Particularly, three human metastatic cancer cell lines were analyzed, namely human mammary adenocarcinoma cells (MDA-MB-231), human urinary bladder carcinoma cells (T24) and human ovarian adenocarcinoma cells (OVCAR-3). Even though cancer cells were able to transmigrate through the endothelial monolayer w/ or w/o the presence of a BMi microenvironment, the extravasation rate was significantly increased in presence of OD hBM-MSCs compared to acellular collagen matrices for OVCAR-3 (50.9 ± 2.2% vs 12.9 ± 3.6%), MDA-MB-231 (64.3 ± 2.9% vs 17.0 ± 4.7%) and T24 (66.9 ± 3.5% vs 16.0 ± 4.5%) (Figure 4A). Similar observations were obtained when combining extravasation rates in BMi for MDA-MB-231, T24 and OVCAR-3 (Figure 4B, black bars) vs acellular gels (60.2 ± 2.7% vs 15.3 ± 2.2%) (Figure 4B, white bars). Interestingly, significant differences were detected comparing MDA-MB-231 or T24 with OVCAR-3 extravasation rates in the BMi microenvironment (Figure 4A, black bars). In fact, the percentages of transmigrated cells for MDA-MB-231 and T24 were equal to 64.3 ± 2.9% and 66.9 ± 3.5%, respectively, which were significantly higher compared to the OVCAR-3 value of 50.9 ± 2.2%. No differences were shown between cancer cell extravasation rates within control matrices (Figure 4A, white bars).

Furthermore, cancer cells were observed to travel within the matrix after transendothelial migration. Therefore, we quantified these cell displacements and found significantly increased migration distances with the BMi microenvironments compared to the acellular ones (33.54 ± 3.22 μm vs 4.77 ± 0.26 μm) (Figure 4C–4E). If we consider an average length of a cancer cell of about 20 μm it is possible to highlight that for all three cancer types, the extravasated cells remained close to the endothelium in acellular matrix condition (migration distance less than 20 μm) while migration occurred only in the presence of the BMi microenvironment (migration distance more than 20 μm).

Noteworthy, in the BMi T24 migrated significantly more than all other cell lines (39.64 ± 7.45 μm T24, 31.6 ± 3.57 μm MDA-MB-231, 26.55 ± 5.47 μm OVCAR-3) (Figure 4C, black bars). Despite similar extravasation rates for T24 and MDA-MB-231 metastatic cancer cells, these migration data suggest a more aggressive behavior of T24 cancer cells, which were not only able to transmigrate across the endothelium but also to migrate considerable distances into the colonized BMi microenvironment.

## DISCUSSION

In this study, we elucidated some aspects of the complex cellular interactions involved in cancer cells extravasation by examining two different aspects of heterotypic intercellular communication. We first demonstrated how transmigrated breast cancer cells altered their gene expression pattern. Although expression analysis on cancer cells migrating though micron-size constrictions have been previously reported [38], the originality of our study relies on the use of more physiologically-relevant samples, namely performing microarray analysis on cancer cells that have transmigrated through an endothelial monolayer. We used two strategies - gene candidates and GO methods - to interpret the global change of expression experienced by transmigrated cancer cells (Figure 1). We notably found clear markers of late-metastatic stages being upregulated while relatively few early-metastatic stage genes were downregulated. Using GO enrichment analysis, we then observed that the four most significant biological terms describing the upregulated DEGs were vasculature development, blood vessel development, circulatory system development and cardiovascular system development. REVIGO analysis confirmed that circulatory system development is one of the three major superclusters which described best the enriched BP terms (Figure 1C, 1D). We postulate that this enrichment could reflect an increase of cell expression related to their mesenchymal phenotype based on an epithelial-mesenchymal transition (EMT) DEGs selection defined by Groger *et al*. in 2012 [39]. Moreover, we found several genes in common between the EMT-core selection and the upregulated DEGs list. Not only are LOX and MMP2 involved in metastatic progression, but also the clinically relevant genes PTX3, NID2, SPOCK1 correlate with poor prognosis; and TGFB1, encoding the cytokine TGF-β, are all upregulated in our microarrays data.

Comparing the downregulated DEGs with the respective EMT-core list, we found three common genes: SLC7A5 which is used to predict recurrence in estrogen receptor positive breast cancer during therapy [40], the epithelial cell marker EPCAM and the gene FAM169A whose role has not yet been defined. EMT has been extensively described and can be associated not only with early stages of metastasis, but also in late-metastatic stages in playing a role during cancer cell extravasation [13]. Therefore, the expression of genes known to play a role in EMT is consistent with our overall results in which markers of late-metastatic progression (Figure 1B), glycocalyx degradation (Figure 2A) and cell migration (Figure 1C, 1D, left panels) are upregulated in transmigrated MDA-MB-231 cells. Moreover, it has been observed that EMT and proliferation are incompatible processes. Indeed TGF-β, a hallmark of EMT which is overexpressed in our microarrays data, is known to induce genomic instability and mitotic defects leading to binuclear cells and increased division time [41]. Those observations are in good agreement with our data since the main downregulated terms are associated with DNA replication and cell cycle (Figure 1C, 1D, right panels). However, care should be taken since EMT is a more complex system, consisting in a spectrum of intermediary phases rather than a two-states model [42]. In addition, MDA-MB-231 cells naturally present mesenchymal characteristics meaning that the observed upregulation of genes involved in EMT could simply reflect an increase in metastatic capabilities of transmigrated MDA-MB-231 cells.

Going deeper into our microarray dataset analysis, we then investigated the expression of glycocalyx rearrangement proteins. CTC extravasation has been linked with partial disruption of the endothelial glycocalyx [6]. It is known that cancer cells secrete MMPs and other degrading enzymes including heparanase and hyaluronidase which affect components of the EC glycocalyx. Moreover, the inhibition of MMP activity by doxycycline was shown to reduce glycocalyx shedding [43], while degradation of HS and hyaluronic acid induced the release of growth factors or cytokines and impaired glycocalyx function, thus compromising endothelial cell-cell junctions and endothelial homeostasis [44]. In our gene expression data, we observed an upregulation of MMPs, along with members of the ADAM and ADAMTS superfamilies. Among the upregulated MMPs, MMP10 and MMP16 functions remain unclear; however, as part of the metalloproteinase family, we can speculate about their possible involvement on EC surface rearrangement alongside MMP1 and MMP2. Similarly, the direct action of ADAM and ADAMTS proteases on glycocalyx impairment has not been demonstrated. Still, ADAMs are members of the same superfamily of MMPs (metzincin zinc-dependent metalloprotease) and are known to play a role in cell adhesion and rearrangement of cell surface molecules [45], while ADAMTSs are secreted molecules which bind to the ECM. From those observations, we postulate that ADAMs might have the potential to work with other metalloprotease superfamilies such as ADAMTSs and MMPs on rearranging the EC surface coating. We furthermore confirmed experimentally the direct negative impact of MDA-MB-231 cancer cells on EC glycocalyx, showing a less intense lectin staining on ECs when close to cancer cells (Figure 2B).

In order to characterize the role of intercellular cross-talk in promoting cancer cell invasion, notably between CTCs and the secondary metastatic site, we then used an alternative version of the 3D tri-culture microfluidic model previously developed by our group [7]. Using this platform, we assessed the impact of bone-like microenvironment on the ability of different tumor cell types to transmigrate through a monolayer of ECs (Figure 3). To achieve this goal, OD hBM-MSCs were embedded or not in collagen gels lined with the endothelium. The endothelial permeability values measured for both BMi and acellular matrices are in the range of previously found permeabilities with HUVECs monolayers, although significantly lower values were quantified with microvascular networks [22, 46, 47]. Consequently, the different extravasation behavior of cancer cells towards BMi and acellular microenvironments was solely due to molecular factors secreted by OD hBM-MSCs and endothelial cells (potentially conditioned by the presence of the BMi microenvironment) without significant effects due to the mechanical properties as the vascular permeability of the endothelial monolayer.

It is well known that breast cancer metastasizes to the bone generating osteolytic bone metastases. Moreover, urinary bladder carcinoma is commonly reported to generate skeletal metastases [48]. Conversely, metastatic ovarian cancer cells preferentially invade contralateral ovary, omentum and abdominal peritoneum [49]. Using our microfluidic device, we showed that the presence of a BMi microenvironment substantially increases the extravasation capacity of breast, bladder and ovarian cancer cells compared to an acellular matrix (Figure 4). Significant differences in the metastatic behavior of these three cells lines towards bone were nevertheless observed, with bladder cells having the highest extravasation rate and migration distance. Despite OVCAR-3 showing lower values compared to the two other cell lines, it should be highlighted that more than 50% of the cells still transmigrated in the BMi microenvironment while clinical studies described an incidence of only 0.82% for bone metastasis [50]. Based on this observed discrepancy we could speculate that the CTC and secondary organ cross-talk is not the main process hampering the formation of ovarian cancer cell metastasis to bone and that metastatic ovarian cancer cells - although able to migrate towards the BMi microenvironment - may be unable to generate micrometastatic colonies once extravasated. However, care should be taken in comparing clinical results with *in vitro* observations since both the Transwell assay and the BMi microenvironment described in this study cannot fully recapitulate the complex physiological mechanisms of cancer colonization in patients.

In summary, we here reported a comprehensive *in vitro* study that combined transcriptomic analyses and a 3D microfluidic approach to highlight the importance of both CTC-endothelium and CTC-secondary tissue interactions in cancer cell extravasation. The identified genes extend current datasets of potential targets involved in the metastatic progression and in particular in CTC-endothelium interactions. We believe that these transcriptomic data coupled with parametric studies of the process of cancer cell extravasation within physiologically-like microenvironments will be helpful to clarify the critical role of glycocalyx degradation in the context of organ-specific extravasation.

## MATERIALS AND METHODS

### Microfluidic device

Microfabrication details for the poly-dimethyl-siloxane (PDMS, Silgard 184, Dow Chemical) devices were previously described [26]. Inlet and outlet ports were bored using disposable biopsy punches and the PDMS layer was bonded to a cover glass to create microfluidic channels following oxygen plasma treatment. Before starting each experiment, the device channels were incubated with a poly-D-lysine hydrobromide solution (PDL, 1 mg/ml; Sigma-Aldrich) for 4 h at 37°C to promote matrix adhesion. A microfluidic device consisting of two lateral media channels and one central gel channel was adopted in the present study. A postless configuration was used to separate gel and media channels. Gel and media channels were 970 μm wide and 120 μm high.

### Cell culture and osteo-differentiation

hBM-MSCs (Lonza) of passage 9 or lower were cultured in standard flasks in osteogenic medium containing L-ascorbic acid, β-glycerophosphate, cholecalciferol and dexamethasone for at least 10 days to induce osteo-differentiation [7]. Calcium deposition by OD hBM-MSCs was characterized through the Alizarin Red-S assay. Briefly, samples were washed twice with 1× Phosphate Buffered Saline (PBS; Invitrogen) and fixed with ice-cold 70% ethanol (EtOH) for 1 h at room temperature. Once completely dried after EtOH removal, samples were washed with double distilled water (ddH_2_O) and incubated with 80 mM Alizarin Red-S (pH 4-4.2) for 15 min at room temperature. Next, samples were washed with ddH_2_O and PBS to remove the excess of staining and reduce unspecific bindings, respectively. Once completely dried, representative pictures were taken using an inverted optical microscope in bright field mode.

Before cell seeding in microfluidic devices, collagenase type I (Gibco) solution (15 mg/ml) was applied for 10 min to promote cell matrix dissolution; then, cells were trypsinized for 5 min. Green fluorescent protein (GFP)-transfected human umbilical vein endothelial cells (HUVECs, Angio-Proteomie) were cultured in Endothelial Cell Growth Medium (EGM-2). All the experiments were conducted using HUVECs of passage 8 or lower. Human mammary adenocarcinoma cells MDA-MB-231 (American Type Culture Collection (ATCC)), human urinary bladder carcinoma cells T24 (ATCC) and human ovarian adenocarcinoma cells OVCAR-3 (ATCC) were selected for high invasiveness and their ability to metastasize *in vivo*. Cancer cells were cultured in Dulbecco's Modified Eagle Medium (DMEM; Invitrogen) supplemented with 10% Fetal Bovine Serum (FBS; Invitrogen), 1% L-glutamine and antibiotics. All cell cultures were kept in a humidified incubator maintained at 37°C and 5% CO_2_ with the medium replaced daily.

### Bone mimicking (BMi) microenvironment and vascular bed formation in the device

Collagen type I (BD Biosciences) solution (6.0 mg/ml) was prepared with 10x PBS, cell water and 1N NaOH, and embedded with OD hBM-MSCs (1.5 × 10^6^ cells/ml). The cell concentration was optimized by balancing the maximum effect induced by OD hBM-MSCs while limiting the possible gel degradation, based on previous studies [7]. 10 μl collagen solution containing OD hBM-MSCs were injected within the gel channel and incubated at 37°C for 30 min inside humidified chambers to polymerize. Osteogenic medium was added to hydrate the hydrogel and provide cell nutrients. After one day, diluted fibronectin solution (50 μg/ml, Sigma-Aldrich) was introduced in the media channels to form a thin coating; microfluidic devices were incubated for 1 h at 37°C, then fibronectin was aspirated and replaced with EGM-2. Next, medium was aspirated from all the reservoirs and endothelial cells (50 μl, 3.2 × 10^6^ cells/ml) in EGM-2 were introduced into a single media channel to generate a monolayer covering channel walls and gel-liquid interface. One reservoir was injected with 40 μl endothelial cell suspension and 10 μl were injected through the opposite reservoir after 5 min to guarantee a homogeneous cell distribution within the channel. The medium was changed 2 h after cell seeding to remove non-adhered cells and reservoirs scraped to dislodge cell clusters. Cells were cultured for 4 days in EGM-2 before cancer cell injection in order to recreate a BMi microenvironment and form the endothelial monolayer at the gel-liquid interface following previous studies [24, 51]. Control experiments were performed with acellular collagen matrices, following the same protocol.

### Extravasation assay in the device

Cancer cells (150,000 cells/ml) were stained with PKH26 Red Fluorescent Cell Linker Kit (Sigma-Aldrich) and injected within the endothelialized channel. As done for the endothelial cells, 40 μl cell suspension was introduced in a reservoir and additional 10 μl were injected via the opposite reservoir after 5 min. Transmigration of cancer cells across the endothelial layer into the BMi microenvironment was analyzed 24 h after the addition of cancer cells to the devices.

### Immunostaining

Samples in the devices were washed with PBS and fixed with 4% paraformaldehyde (PFA) for 15 min at room temperature. Next, cells were washed twice with PBS and incubated with 0.1% Triton-X 100 solution for 5 min at room temperature. After washing twice with PBS, cells were blocked with 5% bovine serum albumin (BSA) solution in PBS for at least 3 h at 4°C. Vascular endothelial-cadherin (VE-cadherin) was labeled with rabbit polyclonal antibody (Enzo Life Sciences) at 1:200 dilution, collagen IV was labeled with rabbit polyclonal antibody (Abcam, 1:500) and laminin was labeled with mouse monoclonal antibody (abcam, 1:50). Primary antibodies were incubated at 4°C for 24 h. Fluorescently-labeled secondary antibodies (Invitrogen) were used at 1:200 dilution (24 h incubation at 4°C). Cell nuclei were stained with Hoechst (1 μg/ml; Invitrogen) at 1:1000 dilution.

Lectins (Bandeiraea Simplicifolia, Sigma-Aldrich, 2 μg/ml in PBS supplemented with 5% BSA) were used to stain HS molecules and identify the cell glycocalyx. Following fixation with 4% PFA for 15 min at room temperature, microfluidic devices were washed twice with PBS and incubated with 5% BSA solution in PBS overnight at 4°C. Finally, samples were incubated with lectin solution at 4°C for 24 h, washed twice with PBS and imaged through confocal microscopy.

### Confocal imaging and data analysis

All images were captured using a confocal microscope (Olympus IX81) and processed with Imaris software (Bitplane Scientific Software). Imaris tracking algorithms were used for selecting and counting cell nuclei and fluorescently labeled cancer cells within a specific region of interest (ROI). The ROI was defined as a 3D region containing both the gel matrix and the endothelial monolayer interfacing the fluidic channel. The ROI dimensions were 600 μm × 600 μm × 120 μm (height). The extravasation percentages and extravasation distances were measured for each ROI and averaged for at least three ROI per device. At least three devices per condition were used for the data analysis. Cancer cell migration was quantified by considering the distance travelled by extravasated cells from the endothelial monolayer. Imaris software was employed to draw lines forming a 90°angle with the monolayer and reaching each extravasated cell.

### Permeability assay

Vessel permeability was quantified according to a previously described method [46]. Briefly, the medium in all reservoirs was aspirated and one of the two media channels was injected with 30 μl fluorescent dextran (70 kDa, green, Invitrogen) diluted with EGM-2 at a final concentration of 20 μg/ml while the opposite channel was simultaneously filled with EGM-2 only. Dextran concentrations were determined by taking confocal images every 2 min for 10 min once equilibrium was established (i.e. a constant intensity within the dextran channel). Permeability was quantified by measuring the average intensity at the initial and final time points considering a 300 μm × 200 μm ROI which included both dextran channel and adjacent gel region. Permeability was computed according to the following formula:

$$P_{D}=\frac{1}{I_{i}-I_{b}}\left(\frac{I_{f}-I_{i}}{\Delta t}\right)\times w$$

where *I_i,_ I_f_* and *I_b_* represent the initial, final and background average intensities, respectively, *Δt* is the time interval between two captured images and *w* is the endothelialized channel width.

### Extravasation assay in Transwell and RNA extraction

Transwell plates (24-well, 8-μm pore size; Sigma-Aldrich) were used to conduct the extravasation assay to retrieve a sufficient number of cells for RNA extraction and microarray analysis (Figure 1A). Inserts were coated with a solution of 50 μg/ml of fibronectin in EGM-2, incubated at 37°C for 1 h and then washed with EGM-2. After the coating, 100 μl of HUVECs cell suspension at 1 × 10^6^ cells/ml was added to the inserts and the plate was incubated at 37°C. After 2 h, the medium in both chambers was replaced with fresh EGM-2 and changed every 24 h. The plate was incubated at 37°C before cancer cell seeding to form an endothelial monolayer. After 48 h, 100 μl of MDA-MB-231 cancer cells suspension was added to the upper chamber at 0.5 × 10^6^ cells/ml in EGM-2. The plate was incubated at 37°C and the medium in the inserts was changed after 1 h to remove non-adherent cells. The plate was incubated at 37°C and cancer cells were allowed to migrate for 24 h. Cells on the upper surface of the inserts were removed using cotton swabs, whereas cancer cells that migrated to the lower surface were trypsinized and collected. Control cells were represented from the original cell population. The RNA extraction was performed with the PicoPure Isolation Kit (Thermo Fisher Scientific) following the manufacturer's protocol. Each experimental condition was performed in duplicate.

### Microarrays and differential expression analysis

The four final RNA samples were submitted to Origen Labs and processed according to Affymetrix and NuGEN recommended protocol, and Origen Labs Standard Operating Procedures (SOP). The samples were first quality controlled (QC) and then profiled using Affymetrix Human Gene 1.0 ST array (Affymetrix, Santa Clara, CA, USA) enabling the analysis of 28,869 genes. Raw data were background-corrected and normalized using Robust Multiarray Average (RMA) from the R- Bioconductor [52] package “oligo”. The Bioconductor package “arrayQualityMetrics” was then used to assess the quality of the microarrays data. For differential expression analysis, the “Limma” package, which uses linear models to analyze microarrays data, was applied on our samples and produced a rank-ordered list of *p*-values. Differentially-expressed genes were then selected based on the cut-off criteria: *p*-values < 0.01, log fold-change values above 1 for up-regulated genes, and under −1 for down-regulated genes. Finally, the web-based tool DAVID, known for functional and pathway enrichment analysis (DAVID; https://david.ncifcrf.gov/) was used for identification of significantly enriched GO terms, for both up and downregulated genes. REVIGO tool was used to reduce the GO term redundancy through clustering and produce summaries of the representative terms [53].

### Statistics

Extravasation rate and extravasation distance values are shown as mean ± standard error of the mean (SEM). 3 to 6 independent devices were considered for the measurements. Permeability values were calculated as mean ± standard deviation. The comparisons between groups were assessed using unpaired Student's *t*-test or one-way ANOVA. Statistical significance was assumed for *p* < 0.05 (^*^). All statistics were performed with PRISM (GraphPad software).

## SUPPLEMENTARY MATERIALS TABLE





## Abbreviations

ADAM: a-disintegrin and metalloprotease
ADAMTS: a-disintegrin and metalloproteinase with thrombospondin motifs
BMi: bone mimicking
BP: biological process
BSA: bovine serum albumin
CTC: circulating tumor cell
DEG: differentially-expressed gene
ddH2O: double distilled water
DMEM: Dulbecco's Modified Eagle Medium
EC: endothelial cell
ECM: extracellular matrix
EGFR: epidermal growth factor receptor
EGM-2: Endothelial Cell Growth Medium
EMT: epithelial-mesenchymal transition
EtOH: ethanol
FBS: Fetal Bovine Serum
GAG: glycosaminoglycan
GFP: Green fluorescent protein
GO: Gene Ontology
hBM-MSC: human bone marrow derived mesenchymal stem cell
HUVEC: human umbilical vein endothelial cell
HS: heparan sulfate
LAMA4: Laminin Subunit Alpha 4
LOX: Lysyl oxidase
MGP: Matrix-Gla Protein
MMP: matrix metalloprotease
OD: osteo-differentiated
PBS: Phosphate Buffered Saline
PDL: poly-D-lysine hydrobromide solution
PDMS: poly-dimethyl-siloxane
PECAM1: Platelet endothelial cell adhesion molecule 1
PTGS2: prostaglandin G/H synthase 2
PFA: paraformaldehyde
QC: quality control
RMA: Robust Multiarray Average
ROI: region of interest
SOP: Standard Operating Procedures
SPARC: secreted protein acidic and rich in cysteine
