# Supplementary material for: A combined microfluidic-transcriptomic approach to characterize the extravasation potential of cancer cells
Source: Oncotarget. 2018 Nov 16;9(90):36110–25. doi: 10.18632/oncotarget.26306 (PMC6281425; doi:10.18632/oncotarget.26306)
Supplement: Supplementary file 1 [file oncotarget-09-36110-s001.pdf]

A combined microfluidic-transcriptomic approach to characterize the extravasation potential of cancer cells

SUPPLEMENTARY MATERIALS

Supplementary Table 1: All genes of MDA-MB-231 extravasated vs MDA-MB-231 control. See Supplementary_Table_1
